# The relationships among overcommitment, effort-reward imbalance, safety climate, emotional labour and quality of working life for hospital nurses: a structural equation modeling

**DOI:** 10.1186/s12912-023-01355-0

**Published:** 2023-06-15

**Authors:** Hui Yu Liang, Tzu Yi Tseng, Hung Da Dai, Jin Yun Chuang, Shu Yu

**Affiliations:** 1 School of Nursing, National Taipei University of Nursing and Health Sciences, Taipei, Taiwan; 2 Department of Nursing, China Medical University Hsinchu Hospital, Hsinchu, Taiwan; 3 Department of Nursing, Taipei Veterans General Hospital, Taipei, Taiwan; 4 School of Nursing, National Yang Ming Chiao Tung University, Taipei, Taiwan

**Keywords:** Nurses, Quality of life, Work, Occupational health, Reward, Motivation, Safety, Emotional intelligence

## Abstract

**Background:**

Quality of working life (QWL) is a highly important issue for nurses. Nurses with lower QWL tend to have lower job performance and intention to stay. The aim of this study was to apply a theoretical model to examine the structural relationships among overcommitment, effort-reward imbalance (ERI), safety climate, emotional labour and QWL for hospital nurses.

**Methods:**

A cross-sectional study design and a simple random sampling method were used to recruit 295 nurses in a teaching hospital and used a structured questionnaire was used to collect data.

**Results:**

Overall, the nurses’ QWL was moderate. Our theoretical model showed a good model fit. Overcommintment had a significant direct positive effect on ERI (*β* = 0.35, *p* < 0.001) and indirect effects on safety climate (*β* = -0.149, *p* = 0.001), emotional labour (*β* = 0.105, *p =* 0.001) and QWL (*β* = -0.061, *p =* 0.004). Additionally, ERI not only had significant direct effects on safety climate (*β* = -0.42, *p* < 0.001), emotional labour (*β* = 0.30, *p* < 0.001) and QWL (*β* = -0.17, *p* < 0.001) but also indirectly affected QWL through safety climate (*β* = -0.304, *p =* 0.001) and emotional labour (*β* = -0.042, *p =* 0.005). Both safety climate (*β* = 0.72, *p* < 0.001) and emotional labour (*β* = -0.14, *p* = 0.003) showed significant direct effects on QWL. Our final model accounted for 72% of the variance in QWL.

**Conclusion:**

Our results highlight the necessity of improving the QWL of nurses. Policymakers and hospital administrators should develop policies and strategies that encourage nurses to exhibit an appropriate level of commitment, balance effort and reward, establish a climate of safety, and reduce emotional labour to improve the QWL of hospital nurses.

## Background

The nurses constitute the largest group of occupational groups in health care organizations accounting for 59% in health professions [1]. Nurses are expected to play multiple roles in clinical practice, which makes nursing a stressful profession and affects the quality of health care [2]. Excessive workload and poor work conditions may lead to low job satisfaction, job stress and poor quality of working life (QWL) [3, 4]. Studies have shown that for most nurses, QWL is low to moderate levels [5, 6] and is affected by job-related stress, burnout [4, 7], intention to leave and actual departure from the profession [4, 8, 9].

QWL is a measure of an individual’s satisfaction with their working life and their personal feelings about their work environment [10, 11]. The broad concept of QWL includes job-related domains (e.g., home–work interface, job and career satisfaction, job-related stress, control at work, and working conditions) and general wellbeing [12]. To retain talented employees and to ensure that employees perform well, an organization should ensure that employees have high QWL [3, 5]. Therefore, improving the QWL of nurses can help ensure high quality of care and nurse retention.

The effort–reward imbalance (ERI) model was developed by Siegrist [13], and Siegrist and Li [14] proposed that balancing effort and reward involves a reciprocal exchange. An imbalance between effort and reward (e.g., high effort, low reward) and excessive overcommitment (an exaggerated form of job involvement) may lead to adverse health effects and poor employee wellbeing [14, 15], which may affect employee QWL. However, few studies have examined the relationships among the ERI, overcommitment, and QWL of nurses.

The factors influencing the QWL of nurses include organizational commitment [5], emotional labour [16], safety climate [17], health status, social support [18], and work environment [5]. However, few empirical studies have employed a single comprehensive theoretical model to examine overcommitment, ERI, safety climate, emotional labour, and QWL to clarify the relationships among these factors [19]. Obtaining a robust and comprehensive understanding of the factors affecting QWL among hospital nurses, which would be helpful in retaining nurses in the workplace. Therefore, we proposed a theoretical model and then examined the structural model fit of the theoretical model.

### Conceptual theoretical framework

**Fig. 1:**
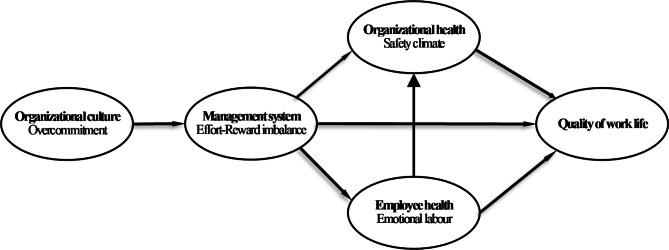
Theoretical model of relationships among overcommitment, effort-reward imbalance, safety climate, emotional labour and quality of working life. (adapted from Peterson & Wilson, 2002, p. 17)

Peterson and Wilson [19] proposed a theoretical model of the culture-work-health model (CWHM) to identify factors influencing QWL. The conceptual framework is composed of organizational culture, management system, organizational health, employee health and QWL. In the CWHM, organizational culture directly affects the management system, which in turn directly affects organizational and employee health and can be a primary determinant of an employee’s QWL. Both organizational health and employee health directly affect an employee’s QWL. Employee health also directly affects organizational health. In this study, we developed a theoretical model based on the CWHM and examined the relationship among overcommitment, ERI, safety climate, emotional labour, and QWL among hospital nurses (Fig. 1). The relevant variables and their relationships with QWL are described in the following sections.

#### Organizational culture

Organizational culture refers to the elements that bind the employees of an organization, such as beliefs, attitudes, behavioral norms and expectations [19]. Organizational culture guides employees’ behavior in organizations [20]. An organization with a strong task orientation may tend to create a high effort or overwork climate for employees in the organization [19]. Overcommitment is defined as a certain characterize with a set of attitudes, behaviors, and emotions reflecting excessive striving at work [13]. Overcommitment seems to characterize an organizational culture that is related to job involvement and is affected by organizations and administrators [21]. Prolonged exposure to overcommitted work tends to create more conflict between work and home, which might result in reduced physical mental health or well-being and poor QWL [14, 22]. Hence, we used overcommitment as a measure of organizational culture and examined its relationship with QWL.

#### Management system

The management system refers to the structure and implementation of management behavior within organizations [19]. Exposure to a higher ERI may lead to higher job-related stress [23]. ERI emphasizes employees’ effort and the reward structure at work. Efforts and rewards also represent extrinsic efforts at work, with expectations to receive certain rewards in return, such as money, esteem, and job security/career opportunities [14]. Rewards are decided by the management system; therefore, this study selected ERI as a variable reflective of the management system and examined its relationship with QWL, particularly in nurses’ populations. The effort-reward (ER) ratio, with effort as the numerator and reward as the denominator, quantifies the amount of ERI. A higher ER imbalance (ratio > 1.0) tends to be associated with work stress and burnout, work-life imbalance and quality of life [24, 25]. Hammig et al. [24] have investigated the relationships between ERI and work-life imbalance, and they found a positive association between ERI and work-life imbalance. Tseng et al. [25] examined the relationships between ERI and quality of life, and found a negative association with physical, mental components. Therefore, we used ERI as the variable of the management system to examine its relationship with QWL. In addition, Kinman and Jones [22] indicated that employees presenting higher overcommitment responded to a lower flexibility of high ERI. In an 8-year longitudinal study, Feldt et al. [26] found that overcommitment was a strong predictor of high ERI, i.e., a high effort but a low reward. Therefore, ERI may play a mediating role between overcommitment and QWL.

#### Organizational health

Organization health refers to the well-being of the corporate whole [19]. Workers’ perception of a safe environment in the workplace tends to have a positive impact on organizational health [19]. Safety climate is defined as employees’ perceptions of safe policies and procedures and feeling of safety in their work environment [27]. Good safety climates have been found to be significantly positively related to employee health outcomes [28]. Nurses perceived safe and nonhazardous in their practice environment tend to present a support and healthy organization [29]. Therefore, we selected safety climate as the variable of organizational health, and we examined its relationship with QWL. Ismail, Asumeng and Nyarko [17] investigated the relationships between safety climate and QWL. In Ghana, a positive relationship existed between safety climate and QWL. Additionally, Peterson and Wilson [19] indicated that organizational health directly affected QWL, that organizational culture had an indirect effect on organizational health through the management system, and that the management system indirectly affected QWL through organizational health. Therefore, in our study, we hypothesized that safety climate may play a mediating role.

#### Employee health

Employee health refers to the well-being of the individual employee [19]. Employees who perceive excessive work demands tend to exhibit negative emotional reactions. Emotional reaction to work stress is a critical component of individual employee health [19]. Emotional labour could be viewed as psychosocial stress. Workers are required to manage their emotions to display behavior that is acceptable to the organization. Frequent emotional displays may result in high emotional labour for nurses, which may lead to job stress and responses with adverse effects on health [14]. Therefore, in our study, we used emotional labour as an indicator for measuring employee health. Cheung and Tang [16] explored the relationships between emotional labour and QWL. They found a negative correlation between surface acting and QWL and a positive correlation between deep acting and the expression of natural felt emotions at work [16]. Peterson and Wilson [19] found that employee health directly affected QWL, that organizational culture had an indirect effect on employee health through the management system, and that the management system indirectly affected QWL through employee health. Therefore, we hypothesized that emotional labour may play a mediating role.

### Hypothesized model

In this study, we obtained the author permission for adopted of the CWHM (Fig. 1) In addition, we proposed a hypothetical model based on the CWHM [19] is as follows: the overcommitment directly affects ERI and indirectly affects safety climate, emotional labour and QWL through ERI. Safety climate and emotional labour directly affect QWL. ERI not only directly affects safety climate, emotional labour and QWL but also indirectly affects QWL through safety climate and emotional labour separately. Moreover, emotional labour directly affects safety climate.

## Methods

### Design, participants, and data collection

A cross-sectional design was employed in this study to explore the relationships among overcommitment, ERI, safety climate, emotional labour and QWL and to test the model fit. Participants were recruited in a 400-bed regional hospital located in northern Taiwan. In this study, using a simple random method was conducted. We identified 533 nurses and used computer-generated numbers to randomly select participants from the 533 nurses. Inclusion criteria were as follow: age being older than 20 years, nurses who are currently full time employed and are willing to participate in the study. Data were collected using a self-administrated questionnaire by participants from January 2018 to July 2018. The sample size was determined by Kline [30] rules, and the minimum sample size needed to be at least 200 for the estimation method in structural equation modeling (SEM). Based on a previous study with a survey study response rate ranging from 66 to 83% [31], we distributed 300 questionnaires. Finally, a total of 295 participants completed the questionnaires; the response rate was 98.3%.

### Measures

We used a structured questionnaire to collect data included: demographics, overcommitment, effort-reward imbalance, safety climate, emotional labour and QWL. Demographics included gender, age, education, marital status, years of nursing, clinical ladder level and work unit.

#### Overcommitment

The 6-items of the Chinese version of the Overcommitment Scale [32] to measure nurses’ overcommitment. Items in this unidimensional scale were rated using a 4-point Likert scale ranging from 1 (strongly disagree*)* to 4 (strongly agree*)*; Total scores range from 6 to 24; a higher score indicated that nurses perceived a higher overcommitment to work. In the present study, the Cronbach’s α was 0.76.

#### Effort-reward imbalance

Two dimensions of the Chinese version of the Effort-Reward Imbalance Scale [32] was used to measure ER imbalance. Respondents scored on a 4-point Likert scale from 1(strongly disagree) to 4 (strongly agree). The ER ratio was calculated based on effort (numerator) and reward (denominator) to quantify the amount of ERI, with the ERI increasing with increasing ratio values. ER = 1 indicated that the nurse reported one effort for one reward, ER < 1 indicated that the nurse reported less effort for each reward, and ER > 1 indicated that the nurse reported more effort for each reward. In the present study, the Cronbach’s α of the ERI scale was 0.74.

#### Safety climate scale

Safety climate was measured used the translated Chinese version [33] and modified versions [34] of the Safety Attitudes Scale was used to assess nurses’ perception of the safety climate status in institutions. Respondents scored on a 5-point Likert scale from 1 (strongly disagree) to 5 (strongly agree) and contains five domains comprising 26 items: teamwork climate (6 items), safety climate (7 items), job satisfaction (5 items), perceptions of management (5 items), and working conditions (3 items). Total scores range from 26 to 130; a higher score indicates a safer climate in the workplace. The Cronbach’s α of the scale was 0.91.

#### Emotional labour

The Chinese version of the Emotional Labour Scale (ELS) [35] was used to measure the emotional effort required in the workplace. The ELS is scored on a 6-point Likert scale ranging from 1 (minimal effort) to 6 (maximal effort) and contains three domains comprising 26 items: expressing positive emotions (5 items), suppressing negative emotions (10 items), and handling others’ negative emotions (11 items). Total scores range from 26 to 156, with a higher score indicating an increase in emotional labour. In the present study, the Cronbach’s α was 0.97.

#### Quality of work life

The Chinese version of the Work-Related Quality of Life Scale (C-WRQoL) was translated and validated by Dai et al. [12] The scale was classified into six domains comprising 23 items: general wellbeing (6 items), home-work interface (3 items), stress at work (2 items), job and career satisfaction (6 items), control at work (3 items), and working conditions (3 items). Scored on a five-point Likert scale ranging from 1 (strongly disagree) to 5 (strongly agree). Total scores range from 23 to 115 and a higher score indicates a higher level of QWL. In the present study, the Cronbach’s α was 0.92.

### Ethical approval

The study was approved by the Institutional Review Board of National Yang-Ming University Hospital (approval number: 2017A032). The information packet received by participants included the purpose of the study, the questionnaire and informed consent. All participants were voluntarily participating was free to stop participating at any time. Participants completed the questionnaire and questionnaire responses were anonymous and confidentiality.

### Statistical analysis

Statistical analysis was carried out with SPSS for Windows version 22.0 (SPSS Institute, Inc., Chicago, IL, USA) software. The frequency, percentage, mean and standard deviation (SD) were used to describe the distribution of participants’ demographic data, overcommitment, ERI, safety climate, emotional labour and QWL. Pearson’s correlation coefficient were used to analysis correlations between variables. SEM was performed using AMOS (version 22.0) software with a two-step modeling approach to test the theoretical hypothesized model [36]. In the first step, a confirmatory factor analysis (CFA) and maximum likelihood estimation (ML) were used to estimate the adequacy of the measurement model. To evaluate whether the concepts were suitable as constructs, we used an indicator of squared standardized outer loadings, and a value > 0.4 was determined; if the value was less than 0.4, the reflected indicator could be deleted [37]. Modification indices (MIs) were used to model modification and identify the relations among the observed and latent variables in the model [38]. In the modified measurement model, the fit indices of the observed variables were significant for the latent variables of overcommitment, ERI, safety climate, emotional labour and QWL.

In the second step, the full structural model was examined to determine whether the hypothesized model fit the present data. The model fit was evaluated using guidelines for fit indices, with acceptable values of fit indices determined based on those recommended by Hu and Bentler [39]. Measurements of the goodness of fit of the proposed theoretical model and the data included χ^2^ (with associated degrees of freedom and *p* value), relative χ^2^ (*χ*^*2*^/*df* < 5.0), root mean square error of approximation (RMSEA < 0.06), goodness-of-fit index (GFI > 0.90), adjusted GFI (AGFI > 0.90), and comparative fit index (CFI > 0.90). Finally, R^2^ was used to present the predictive power of the model. To test the statistical significance of the indirect effect of the proposed model, we used the bootstrapping method with 2000 resamples, and bias-corrected 95% confidence intervals (CIs) were implemented to test indirect effect [40].

## Results

### Characteristics of the nurse participants

In total, 295 nurses participated in this study (Table 1). The mean age of the participants was 32.42 (SD = 6.91); 64.7% were younger than 35 years. The majority (97.3%) of the participants were female; 73.6% of participants held a baccalaureate; and 50.2% were unmarried. The mean number of years of nursing work experience was 9.66 years (SD = 6.79); most of the participants’ clinical ladder level was the N2 level (43.1%), and 34.5% of the participants worked in wards (including medical, surgical and gynecology/pediatric wards).

**Table 1:** Demographics of participants (*N* = 295)

Variables	n	%	*M* (*SD)*	Range
**Age**			32.42 (6.91)	21.08–51.17
< 24 years	51	17.3		
25–34 years	140	47.4		
35–44 years	91	30.9		
> 45 years	13	4.4		
**Gender**				
Male	8	2.7		
Female	287	97.3		
**Educational level**				
Associate degree	73	24.7		
Bachelor’s degree	217	73.6		
Graduate degree	5	1.7		
**Marital status**				
Unmarried	148	50.2		
Married	130	44.1		
Other	17	5.8		
**Years of nursing**			9.66 (6.79)	0.08-30.00
0–1 1–5 6–10 11–15 16–20 21^+^	15 92 72 60 35 21	5.1 31.2 24.4 20.3 11.9 7.1		
**Clinical ladder level**				
N, N0	57	19.3		
N1	82	27.8		
N2	127	43.1		
N3	26	8.8		
N4	3	1.0		
**Work of units**				
Medical	70	23.7		
Surgical	16	5.4		
GYN/PED	13	4.4		
OR	36	12.2		
ER/ICU	89	30.2		
OPD	25	8.5		
Community health	27	9.2		
Other	19	6.4		

*Note.* M; mean, SD; standard deviation; GYN/PED, gynecology/pediatric; OR, operating room; ER, emergency room; ICU, intensive care unit; OPD, outpatient department

### Descriptive statistics of major variables

As shown in Table 2, the average overcommitment score was 2.75 (SD = 0.49), indicating that nurses had a slightly lower degree of overcommitment. Regarding ERI, nurses reported a slight ER imbalance (ER ratio = 1.05, SD = 0.23), with a slightly higher degree of effort than reward. In addition, nurses perceived a moderate degree of safety climate (M = 3.53, SD = 0.41) and emotional labour (M = 3.46, SD = 1.07) in their work environment. Furthermore, the overall average score for QWL was 3.26 (SD = 0.44), indicating a moderate degree of QWL. Among the six dimensions of QWL, stress at work showed a lower degree, while the other five dimensions were ranked as moderate degree, with mean scores ranging from 3.19 to 3.43. The highest mean subscale scores were observed for control at work (M = 3.43, SD = 0.54) and job and career satisfaction (M = 3.41, SD = 0.47); the lowest mean subscale score was observed for stress at work (M = 2.60, SD = 0.68).

**Table 2:** Descriptive statistics of variables (*N* = 295)

Variables	Mean	SD	Range
**Overcommitment**	**2.75**	**0.49**	**1–4**
**Effort-reward imbalance**	**1.05**	**0.23**	
Effort	3.01	0.47	1–4
Reward	2.92	0.34	1–4
**Safety climate**	**3.53**	**0.41**	**1–5**
Teamwork climate	3.39	0.45	1–5
Safety climate	3.61	0.50	1–5
Job satisfaction	3.44	0.63	1–5
Perceptions of management	3.49	0.53	1–5
Working conditions	3.61	0.61	1–5
**Emotional labour**	**3.46**	**1.07**	**1–6**
Handling others’ negative emotions	3.36	1.16	1–6
Expressing positive emotions	3.16	1.16	1–6
Suppressing negative emotions	3.71	1.15	1–6
**Quality of working life**	**3.26**	**0.44**	**1–5**
General well-being	3.19	0.55	1–5
Home-work interface	3.32	0.63	1–5
Job and career satisfaction	3.41	0.47	1–5
Stress at work	2.60	0.68	1–5
Control at work	3.43	0.54	1–5
Working conditions	3.28	0.58	1–5

*Note.* SD; standard deviation

### Measurement model analysis

CFA was employed to test the measurement model for latent constructs; the model fit was tested for observed and latent variables among constructs. We examined the indicator outer loadings to determine the suitability of the concepts as constructs; the findings indicated that as initially determined, the observed variable “stress at work” had a value of 0.11 for the QWL. The removal of dimension variable resulted in a modified measurement model with a good model fit for the QWL measurement model (χ^2^ = 309.01 =, *df* = 163, *p* < 0.001, RMSEA = 0.05, GFI = 0.93, AGFI = 0.90, CFI = 0.95).

### Correlation of major variables

Table 3 shows the relationships between major variables. QWL was found to be significantly positively associated with safety climate (*r* = 0.613, *p* < 0.001) and significantly negatively associated with overcommitment (*r* = -0.291, *p* < 0.001), ERI (*r* = -0.531, *p* < 0.001), and emotional labour (*r* = -0.310, *p* < 0.001).

**Table 3:** Correlation coefficients of variables

Variables	1	2	3	4	5
1. Overcommitment	1				
2. Effort-reward imbalance	0.353^**^	1			
3. Safety climate	0.031	-0.334^**^	1		
4. Emotional labour	0.260^**^	0.310^**^	-0.013	1	
5. Quality of working life	-0.291^**^	-0.531^**^	0.613^**^	-0.310^**^	1

Note.^**^p < 0.001

### Structural model fit and hypotheses test

**Fig. 2:**
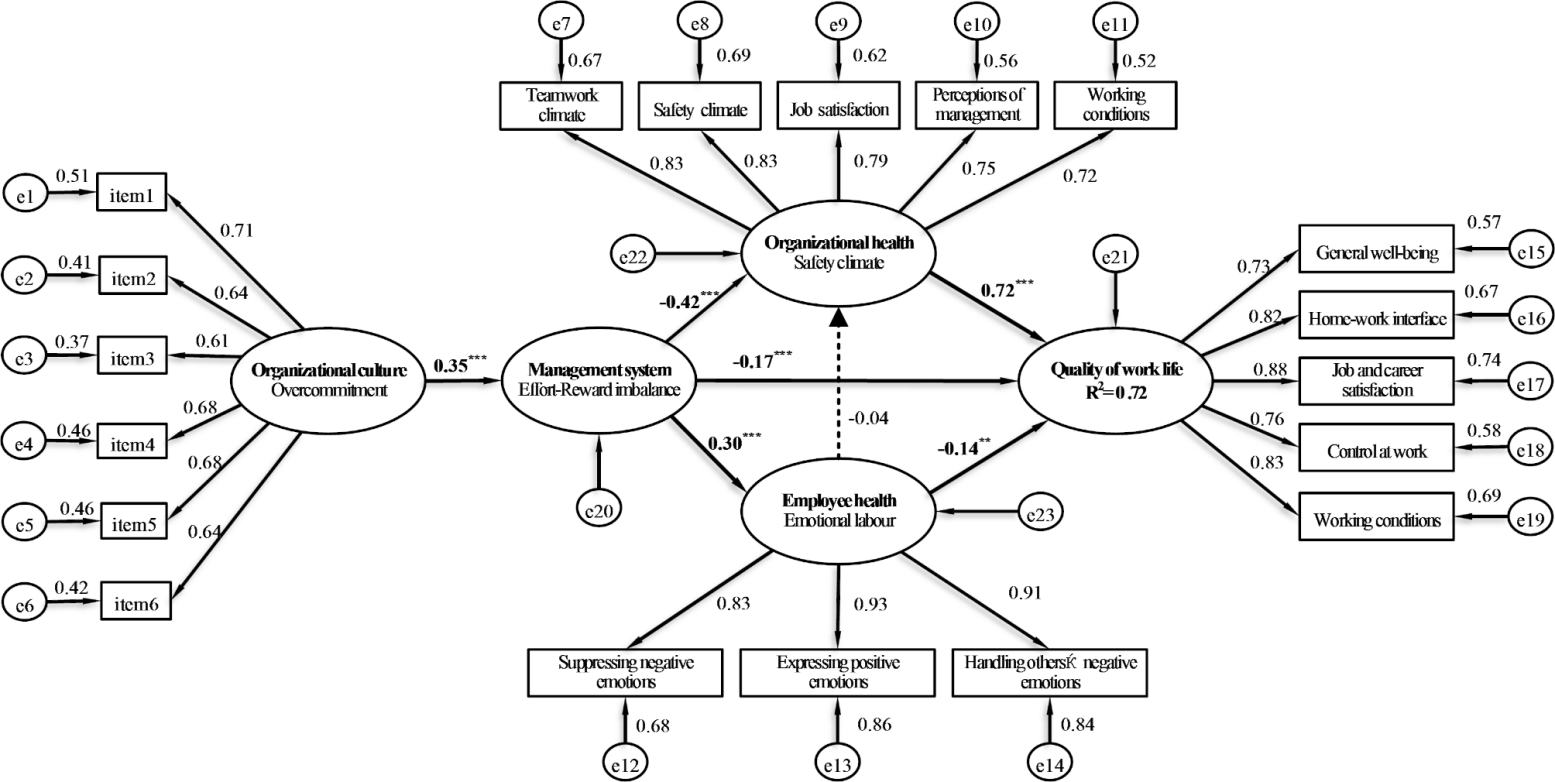
Model of the relationships among the latent variables of overcommitment, effort-reward imbalance, safety climate, emotional labour and quality of working life among nurses. The data presented as the latent variables are standardized path coefficients. Solid lines indicate that the path is statistically significant; dashed lines indicate that the path is not statistically significant. Note.^*^p < 0.05, ^**^p < 0.01, ^***^p < 0.001

SEM was employed to examine the fit of the structural model, and the findings revealed a good model fit (χ^2^ = 142.89, *df* = 73, *p* < 0.001; χ^2^/*df =* 1.96, RMSEA = 0.057, GFI = 0.94, AGFI = 0.90, CFI = 0.98). Figure 2 shows that the final model and findings accounted for 72% of the variance in QWL. For the hypotheses test, the proposed hypotheses were partial supported. Regarding direct affects, overcommitment had a significant positive direct effect on ERI (*β* = 0.35, *p* < 0.001), indicating a higher level of overcommitment to work with a greater ERI. ERI had a negative direct effect on QWL (*β* = -0.17, *p* < 0.001), indicating that the higher the level of ER imbalance (ratio > 1.0) was the lower the QWL. Furthermore, ERI also had a negative direct effect on safety climate (*β* = -0.42, *p* < 0.001) and a positive direct effect on emotional labour (*β* = 0.30, *p* < 0.001). Nurses perceived a higher level of ERI, revealing a lower level of safety climate and a greater level of emotional labour. Safety climate had a strongly positive direct effect on QWL (*β* = 0.72, *p* < 0.001), indicating that a higher level of safety climate was associated with better QWL. Emotional labour had a negative direct effect on QWL (*β* = -0.14, *p* = 0.003), meaning that nurses who experienced a higher level of emotional labour had lower QWL. However, no significant effect was observed between emotional labour and safety climate (*β* = -0.004, *p* = 0.532).

With respect to indirect effects (Table 4), overcommitment had a significant negative indirect effect on safety climate (*β* = -0.149, *p* = 0.001) through ERI, indicating that ERI played a mediating role between overcommitment and safety climate. Overcommitment revealed a significant positive indirect effect on emotional labour (*β* = 0.105, *p* = 0.001) through ERI, meaning that ERI played a mediating role between overcommitment and emotional labour. Furthermore, the negative indirect effect of overcommitment and QWL was significant (*β* = -0.061, *p* = 0.004); therefore, ERI also mediated the relationship between overcommitment and QWL. In term of ERI, there are had a negative indirect effect on QWL (*β* = -0.304, *p* = 0.001) through safety climate, indicating that safety climate played a mediating role between ERI and QWL. Moreover, ERI also revealed a negative indirect effect on QWL (*β* = -0.042, *p* = 0.005) through emotional labour; therefore, emotional labour played a mediating role between ERI and QWL.

**Table 4:** Indirect effects of the path model

Model pathways					β	*p*	95% confidence interval	
							Lower bounds	Upper bounds
**Indirect effect**								
Overcommitment	→	ERI	→	Safety climate	-0.149	0.001	-0.217	-0.092
Overcommitment	→	ERI	→	Emotional labour	0.105	0.001	0.055	0.173
Overcommitment	→	ERI	→	QWL	-0.061	0.004	-0.127	-0.019
ERI	→	Safety climate	→	QWL	-0.304	0.001	-0.413	-0.209
ERI	→	Emotional labour	→	QWL	-0.042	0.005	-0.084	-0.009

*Note.* ERI: Effort-reward imbalance; QWL: Quality of working life

## Discussion

Quality of working life could allow employees to be effectively retained in the workforce and ensure the quality of performance. Our results revealed that nurses had a moderate level of QWL. The findings are similar to those of Akter et al. [5], Alharbi et al. [41], Zandian, Sharghi and Moghadam [42] and Dai et al. [12]. The results remind us that it is necessary to develop more effective strategies to promote nurses’ QWL. Among the six dimensions, the findings showed that the highest mean score was for “control at work and job”, while the lowest score was for “stress at work”. This finding is dissimilar to that of Dai et al. [12] using the same scale; the authors found that the highest score was for “job and career satisfaction”. The reason for this inconsistency may be differences in clinical ladder level. In the study of Dai et al. [12], there were 75.5% more nurses with an N2 or above clinical ladder level higher than our sample. Nurses with a higher clinical ladder level may have more opportunities to grow in their careers and be more satisfied with their jobs [43].

In the present study, we employed our theoretical model to examine the factors influencing the QWL of nurses. SEM analysis revealed a good fit of the structural model, which accounted for 72% of the variance of QWL. Model fit statistics confirmed that variables described in partially support our hypotheses affected nurses’ QWL. Our findings showed that overcommitment had a direct positive effect on ERI; this finding is similar to that of Feldt et al. [26]. Employees who perceive a high degree of overcommitment tend to invest much effort in their work but do not obtain adequate rewards [26]. Thus, overcommitment results in a higher ERI (ratio > 1.0). Consistent with the hypothesis, ERI negatively affected QWL. Employees encountering higher ERI experience more psychological stress and exhaustion [10], which may lead to increased dissatisfaction in terms of job well-being [44] and lower QWL. Therefore, ERI negatively affects QWL. Our findings also confirmed that ERI had a direct negative effect on safety climate. This finding is consist with that of Phipps, Malley and Ashcroft [45], who found that employees exerted an adequate level of effort, and that the existence of rewards could facilitate safety-related behavior [45]. A higher ERI imbalance led to higher work stress associated with poorer psychosocial care and increased errors [46] and could negatively affect nurses’ perceived safety climate.

The present study, ERI positively directly affected emotional labour. This finding is similar with that of de Jonge et al. [47]. When nurses perceive higher efforts and low rewards in their work, this may increase negative psychological reactions and lead to high emotional labour. Among the variables, safety climate had the strongest positive direct effect on QWL. This finding is consistent with that of Ismail, Asumeng and Nyark [17], who found that workers who perceived a positive safety climate were likely to experience high QWL. The present study indicated that emotional labour negatively directly affected QWL. This finding is consistent with Cheung and Tang [16]. Nurses need to frequently display surface acting emotion, which might lead to job stress [16]. Higher job stress may result in poor QWL.

This study found that emotional labour did not influence the safety climate and hypothesis was not supported. This finding is similar to that obtained by Liang et al. [34] and Lee et al. [48]. The possible reasons may be related to the safety climate as a commitment to safety issues [49]. Safety practices in daily work and safety improvements were regarded as parts of a nurse’s role [45]. In addition, emotional labour is an intrinsic aspect of the nurse’s work role [50]. Our path coefficients indicated that organizational safety climate had a stronger effect than did personal emotional labour. Therefore, even when the nurses perceived a higher level of emotional labour at work, they still maintained safety practices. That is, emotional labour did not significantly affect safety climate.

Overcommitment revealed an indirect negative effect on safety climate mediated by ERI. The findings are similar to those of Phipps et al. and Lee et al. [45, 51]. Employees who perceive a higher overcommitment at work may neglect their safety improvement and safety work behaviors [45, 51]. As mentioned earlier, overcommitment is an antecedent of ERI, and nurses who engage in higher overcommitment in their work environments tend to increase investments in effort into the work, resulting in higher ERI, which may constrain their ability to make timely safety improvements and cause them to engage less in safe work behaviors [51]. Therefore, ERI plays a mediating role between overcommitment and safety climate.

Our findings indicated that overcommitment had an indirect positive effect on emotional labour through ERI. This finding is consistent with that reported by de Jonge et al. [47], who discovered that employees who exhibited characteristics of overcommitment and perceived a larger ERI imbalance (high effort and low reward) were 21 times more likely to experience emotional exhaustion. Nurses tend to exhibit overcommitment, a strong need for control, and difficulties relaxing after work. Nurses with a higher level of overcommitment tend to perceive a larger ERI imbalance, leading to negative emotions [52]. Thus, ERI plays a mediating between overcommitment and emotional labour.

In present study, we first found that overcommitment had an indirect negative effect on QWL through ERI. Organizations exhibit particularly high work demands and are characterized by excessive commitment to work environments. Employees experience a higher imbalance in their work-life balance, which may result in poorer QWL [44]. Furthermore, according to path analysis, ERI mediated the relationship between overcommitment and QWL. Nurses who perceive high work demands and excessive commitment in the workforce tend to have a higher imbalance between effort and rewards, resulting in a decrease in their work-life quality.

In this study, ERI not only revealed a direct negative effect on QWL but also indirectly affected QWL through safety climate and emotional labour. Nurses who perceive a higher ERI may reduce safety-related behavior and increase errors, making them more likely to have lower QWL [45]. Therefore, safety climate plays a mediating role between ERI and QWL. With respect to emotional labour, as mentioned earlier, nurses with higher levels of ERI are more likely to have increased job stress [16], resulting in negative emotional well-being [47] and reducing QWL. Thus, when nurses perceive a higher ERI, they are more likely to have a negative emotional reaction, leading to poor QWL.

## Conclusions

QWL is important for retaining employees and ensuring the quality of performance in the workforce. However, our study found that nurses had a moderate level of QWL overall. The highest score was noted for “control at work”, and the lowest score was found for “stress at work”. Therefore, we recommend that more effective strategies be developed to enhance hospital nurses’ QWL, particularly in decreasing job-related stress. This study proposed a theoretical model for the factors influencing the QWL of nurses (i.e., overcommitment, ERI, safety climate, and emotional labour) and tested the fit of the theoretical model. The findings of this study reveal that the model had a good fit and therefore can effectively illustrate how these factors affect QWL among nurses. Our study provides support for the following hypothesis that QWL would be directly affected by ERI, safety climate, and emotional labour. Overcommitment not only directly affects ERI but also indirectly affects safety climate, emotional labour, and QWL. ERI has indirect effects on QWL through safety climate and emotional labour. Our findings reveal that appropriate levels of commitment, a low ERI, low emotional labour levels, and a highly safe climate can improve hospital nurses’ QWL both directly and indirectly. Because low levels of overcommitment can indirectly improve hospital nurses’ QWL, we recommend that hospital managers focus on establishing a work culture in which overcommitment is discouraged. In addition, ERI is a key influencer of safety climate, emotional labour, and QWL. Providing a reasonable number of rewards can ensure a balance between effort and reward, improve the safety climate, reduce emotional labour, and improve the QWL of nurses. We also recommend that nurse administrators develop strategies to help nurses improve their emotional skills, thereby enabling them to effectively communicate with patients, reducing job-related stress, and increasing their QWL. Additionally, policymakers and managers should provide safety training programs to improve nurses’ safety attitudes and ensure supportive and safe work environments, thereby increasing nurses’ QWL.

### Strengths and limitations

The present study has several strengths and limitations. A strength of this study is that it employed a theoretical model to examine how overcommitment, ERI, safety climate and emotional labour contribute to the QWL of hospital nurses. Crucially, the model was tested using scientific methods to assess model fit.

Despite the study’s strengths, our study had some limitations. First, the research setting was a teaching hospital. A multicenter study is recommended for future research to obtain results that are generalizable to other hospitals. Second, an increase in sample size also be considered for future research might provide a more accurate generalization of the study results to other hospitals. Finally, the use of qualitative methods such as triangulation method is recommended to gain a more comprehensive understanding of the relationships between relevant variables.

## Data Availability

The datasets used and/or analysed during the current study are available from the corresponding author on reasonable request.

## Abbreviations

QWL: Quality of Working Life
ERI: Effort-Reward Imbalance
CWHM: Culture-Work-Health Model
ELS: Emotional Labour Scale
C-WRQoL: Chinese Version of the Work-Related Quality of Life
SD: Standard Deviation
SEM: Structural Equation Modeling
ML: Maximum Likelihood
CFA: Confirmatory Factor Analysis
MIs: Modification Indices
RMSEA: Root Mean Square Error of Approximation
GFI: Goodness-of-Fit Index
AGFI: Adjusted Goodness-of-Fit Index
CFI: Comparative Fit Index
CIs: Confidence Intervals
